# Ultrasound Microscopy-Based Identification of Enamel and Restorative Materials: An Ex Vivo Acoustic Impedance Study

**DOI:** 10.1016/j.identj.2025.100880

**Published:** 2025-06-30

**Authors:** Yukihiro Naganuma, Masatoshi Takahashi, Yoshifumi Saijo, Masahiro Iikubo, Atsushi Takahashi

**Affiliations:** a Clinics of Dentistry for Disabled, Tohoku University Hospital, Sendai, Miyagi, Japan; b Division of Biomaterials and Bioengineering, School of Dentistry, Health Sciences University of Hokkaido, Ishikari, Hokkaido, Japan; c Biomedical Imaging Laboratory, Graduate School of Biomedical Engineering, Tohoku University, Sendai, Miyagi, Japan; d Division of Dental Informatics and Radiology, Tohoku University Graduate School of Dentistry, Sendai, Miyagi, Japan

**Keywords:** Hardness, Acoustic impedance, Composite resins, Glass ionomer cements, Dentistry, Dental enamel

## Abstract

**Introduction and aims:**

Differentiating restorative materials from enamel during dental examinations is challenging because of their similar appearances. Even with ultrasound microscopy, the acoustic properties of restorative materials remain unassessed. This study investigated the potential of ultrasound microscopy to differentiate between enamel, composite resin, and glass ionomer.

**Methods:**

Extracted third molars served as the tooth model: a 1.2 mm-diameter cylindrical cavity drilled into the enamel and restored with either composite resin (flowable bulk-fill or paste) or glass-ionomer cement (conventional, high-filler, or multi-ion). To evaluate the restorative materials, a second model was prepared by milling a 2.0 mm-diameter, 3.0 mm-deep cavity into a PMMA block and filling it with the same materials. Both models were imaged with ultrasound microscopy to obtain acoustic-impedance maps, and the PMMA specimens subsequently underwent Vickers hardness testing to explore the correlation between hardness and acoustic impedance.

**Results:**

Acoustic impedance was measured with an accuracy of 16 µm × 16 µm per pixel over an area of 4.8 mm × 4.8 mm, allowing for the construction of 2-dimensional colour images that effectively differentiated between enamel and restorative materials. The colour distribution for CR was homogeneous, while GIC exhibited a heterogeneous distribution across all samples. The mean acoustic impedance of enamel (15.6 ± 4.37 kg/m²s) was significantly greater than that of CR (Type Flow 5.36 ± 0.264 kg/m²s, Type Paste 5.49 ± 0.323 kg/m²s) and GIC (Type high-filler 4.80 ± 0.360 kg/m²s, Multiple ion 3.80 ± 0.360 kg/m²s, Conventional 3.74 ± 0.353 kg/m²s) (*P* < .01). A distinct threshold was established based on the combined standard deviations (σ₁ + σ₂). Pairwise comparisons confirming the distinguishability of enamel, CR, and GIC.

**Conclusion:**

Ultrasound microscopy effectively distinguishes between enamel and restorative materials, as well as between restorative materials (CR and GIC) through acoustic impedance measurement.

**Clinical relevance:**

These findings suggest that ultrasound microscopy may assist in identifying restoration margins and assessing materials in clinical settings.

## Introduction

Composite resins (CR) and glass-ionomer cements (GIC) are the most commonly used materials for direct restoration of small and partial carious defects.^1^

CR and GIC possess distinct properties. CR can be classified into chemically- and light-cured types, achieving adhesion through etching, priming, and bonding processes. They are recognised for their high adhesiveness and colour matching with natural teeth.^2^^,^^3^ In contrast, GICs adhere directly to tooth structures via ionic and hydrogen bonding and are noted for their sustained fluoride release, which aids in the prevention of caries.^4^

Special-care dentistry often faces severe patient-management limitations that make optimal isolation and extended chair-time challenging. In these circumstances, minimal-intervention techniques, such as atraumatic restorative treatment (ART) and interim therapeutic restorations (ITR), are recommended, both of which rely heavily on GICs because of their chemical adhesion, fluoride release, and tolerance to moisture.^4^, ^5^, ^6^, ^7^, ^8^ Consequently, GICs are the material of choice for definitive or temporary restorations in many special-care clinics, even for lesions that would typically receive CR in cooperative patients. This clinical reality justifies evaluating multiple GIC formulations alongside CRs when assessing any diagnostic modality intended for universal chairside use.

Although conventional GICs, such as Fuji VII, are known to have inferior translucency and colour-matching properties compared to CRs–rendering them less suitable for highly esthetic zones such as the anterior teeth. They are still considered restorative materials due to their tooth-coloured shades (eg, off-white or pale yellow), which are more aesthetically acceptable than metallic alternatives. Furthermore, in special care dentistry, where patient cooperation is often limited, Fuji VII is commonly used in ART and ITR procedures because of its chemical adhesion, fluoride release, and ease of handling. This underscores its relevance as a restorative option in minimally invasive treatment contexts.

The longevity of direct restorations is limited; replacement procedures account for approximately 50% to 70% of all operative dentistry in adults.^9^^,^^10^ During retreatment, failure to identify the existing restorative material can lead to over-preparation, incomplete removal, or inappropriate material selection, thereby compromising the prognosis and chair-time efficiency. This risk is not limited to restorative failure; misinterpretation of dental charts caused by visual misidentification may also hinder personal identification in forensic odontology or disaster victim identification (DVI), where accurate dental records are essential.^11^

Despite their advantageous properties, differentiating between CR and GIC restorations during visual inspection presents challenges. Both materials exhibit colour matching with natural teeth, complicating the identification of restorations, particularly under varying lighting and oral hygiene conditions. This difficulty has implications for accurate diagnosis and treatment planning, emphasising the need for effective differentiation methods.^2^, ^3^, ^4^ Recent *ex vivo* investigations have quantified this problem: conventional operatory-light inspection identified composite margins with sensitivities of 36% and 27%, respectively, even when performed by experienced clinicians.^12^^,^^13^

Ultrasound microscopy has been proposed as a novel dental diagnostic device.^14^ It employs ultrasonic waves propagating through a medium, such as water, to visualise the acoustic properties of an object by analysing the reflected and transmitted ultrasound waves directed at a localised region. The obtained acoustic parameters maintain a constant relationship with the tissue elastic modulus, which reflects material rigidity; thus, an ultrasound microscope can effectively evaluate biomechanics. Additionally, it allows real-time observation of tissues without necessitating staining or invasive procedures.^15^, ^16^, ^17^, ^18^ This technology is primarily utilised within the medical field to diagnose soft tissue diseases, such as conditions affecting the cardiovascular system and articular cartilage, *in vivo*. Currently, 3-dimensional colour imaging is employed to assess various pathological conditions.^19^^,^^20^ Recently, significant advancements have been made in miniaturising peripheral equipment and developing transducers for clinical applications.^21^ A previous investigation by the present research group assessed the acoustic characteristics of surrounding dentin in cavities using this technology; it revealed a notable difference in acoustic characteristics between carious and healthy dentin, supporting the efficacy of ultrasound microscopy in evaluating caries extent.^14^ Furthermore, the results obtained were correlated with Knoop hardness—utilised as a criterion for tooth preparation and caries removal—underscoring the clinical relevance of measuring acoustic characteristics with an ultrasound microscope.^14^

As previously mentioned, restorative materials are expected to fulfil esthetic requirements by replicating the colour of natural teeth; however, this often complicates the accurate differentiation between tooth structure and restorative materials through visual inspection alone. Such limitations may lead to misidentification of restoration margins, incorrect evaluation of restoration integrity, and challenges in treatment planning. Additionally, inadequate differentiation between natural tooth structure and restorative materials can impact clinical decisions regarding retreatment or further restorative interventions. To date, no study has evaluated the acoustic properties of dental restorative materials employed in clinical practice.

To address these challenges, this study evaluated whether ultrasound microscopy can discriminate enamel from representative restorative materials—CR and GIC— during retreatment, thereby supporting clinical decision-making.

## Materials and methods

### Ethical considerations

Approval for this study was obtained from the Ethics Committee of the Tohoku University Graduate School of Dentistry (Approval No. 2019-3-37). Patients scheduled for extraction of third molars deemed unrestorable due to pericoronitis at the Tohoku University Graduate School of Dentistry were included in the study. Third molars were selected for practical and ethical reasons: their extraction is the first-line treatment for pericoronitis or impaction and therefore imposes no additional risk or inconvenience on the patients, while simultaneously providing a sufficient number of sound specimens under a single institutional review board approval. The purpose of the study was explained to the patients, and extracted third molars were collected from those who provided informed consent. All participants were fully informed about the study procedures and their right to withdraw at any time without consequences. Written informed consent was obtained from all participants prior to inclusion in the study. All experiments on human subjects were conducted in accordance with the Declaration of Helsinki (http://www.wma.net).

### Tooth-based model

This tooth-based model was used to evaluate the enamel–restoration interface using both qualitative imaging and quantitative measurements. The crowns and roots of extracted teeth were sectioned using a diamond disk and the crowns were embedded in a room-temperature polymerising resin (SPLINT RETAINER RESIN, GC, Tokyo). The embedded samples were subsequently sectioned using a Low-Speed Diamond Wheel Saw (South Bay Tec., Canada) and polished until a smooth surface was achieved. A cavity with a diameter of 1.2 mm was prepared in the enamel using a diamond bur, which was subsequently filled with CR or GIC. The commercial names, classifications, manufacturers, and other relevant details are summarised in Supplementary Table S1. For CR restorations, a 45° circumferential bevel was prepared around each tooth cavity. A single-component self-etch adhesive (BeautiBond Xtreme; SHOFU, Kyoto, Japan) was subsequently applied in accordance with the manufacturer’s instructions and then light-cured.

For CR, flowable bulk-fill and paste types were selected, while standard, high-filler, and multi-ion types were chosen for GIC. Following the application of the dental materials to the cavity, the restoration surface was meticulously polished to prepare samples for subsequent imaging analysis. The samples were categorised into the following groups: enamel, flowable bulk-fill CR, paste CR, standard GIC, high-filler GIC, and multi-ion GIC. A high-filler esthetic GIC (Fuji IX) and a multi-ion–releasing GIC (Caredyne) were selected to represent contemporary esthetic formulations. A conventional GIC (Fuji VII) was included as the reference material within the GIC family, its marked opacity providing the baseline for acoustic-impedance measurements. Flowable bulk-fill and paste CRs were chosen as the routinely used tooth-coloured restorative comparators.

### Acrylic-based reference model

This separate acrylic block model served to compare restorative materials without tooth tissue. Separate samples were prepared to compare the acoustic impedances of the restorative materials. A cylindrical cavity, 3.0 mm in depth, was created on an acrylic plate measuring 30 mm × 50 mm × 5 mm (Hikari AF501, Osaka, Japan) by utilising a milling machine (PROXXON Micro Milling Table MF70) and an end mill with a diameter of 2.0 mm. The restorative materials were filled into these cavities as previously described, and imaging was conducted using an ultrasound microscope (Supplementary Table S1). The schematic representation of the acoustic impedance measurement of restorative material samples is depicted in Supplementary Figure S1. Previous research on the physical properties of restorative materials was used as a reference,^22^ and to ascertain the requisite sample size for comparing Vickers hardness and impedance values, calculations were performed with a significance level of 0.05, power of 0.8, and an effect size of 0.8 using EXCEL Toukei Ver. 8.0. The required number of samples was thus calculated to be 26 per group.

### Ultrasound microscopic observation

#### Ultrasound microscope system

An ultrasound microscope (AMS-50SI; Honda Electronics, Aichi, Japan) was used in the experiment. A block diagram of the ultrasound microscope system is provided in Supplementary Figure S2. The ultrasound microscope facilitates 2-dimensional scanning with an ultrasound transducer, enabling noninvasive measurement of acoustic parameters of tissue sections and cells at the microscopic level. The acquired information is visualised on the display as clear images. The microscope system consists of 5 units. In this experiment, in accordance with previous research,^14^ the pulse generator was configured to a voltage of 40 V, with a rise time of 400 ps and a pulse width of 2.0 ns. The transducer had a central frequency of 80 MHz, an aperture of 1.8 mm, and a focal length of 2.5 mm. Supplementary Figure S3 displays an enlarged view of the measurement unit. The ultrasound waves are emitted and received by the same transducer.

#### Imaging assessment

Imaging was performed on the samples prepared in Section 2.2 utilising the ultrasound microscope. The observation area encompassed 4.8 mm × 4.8 mm for each measurement. Acoustic impedance data for each pixel were sampled at 1.0 GHz using a digitizer (Tektronix TDS7154B, Beaverton, USA) and subsequently transferred to the system-controlled PC in bulk. The acoustic impedance values acquired were categorised into 256 colour tones ranging from 2.5 kg/m^2^ (blue) to 10.0 kg/m^2^ (red), and displayed as colour images on the monitor.

#### Acoustic impedance measurement

The tooth specimens imaged in Section 2.2 were used directly for acoustic-impedance measurement; no additional tooth samples were prepared. Twenty-six specimens that satisfied the criteria of surface flatness and enamel integrity were included in both the qualitative imaging and quantitative impedance analysis. Only teeth with clearly visible enamel surfaces were selected, and the PMMA samples prepared in Section 2.3 were used to evaluate the restorative materials. For enamel specimens, a visually intact enamel area was first identified at random because the cavity centre was not applicable; the mean acoustic-impedance value was then calculated from a 10 × 10-pixel window centred on that point. For restorative materials embedded in PMMA, the window was centered on the geometric centre of each cylindrical filling. For each material, this procedure was applied to all 26 specimens, generating the final impedance data set.

### Vickers hardness test for restorative materials

Vickers hardness testing was performed on the restorative materials to investigate the relationship between Vickers hardness and acoustic impedance values. The Vickers hardness of the samples specified in Section 2.3 was measured using a microhardness tester (HM-102, Mitutoyo, Japan) equipped with a Vickers indenter. The measurement point was centred on each restorative material, with the loading condition set at 0.245 N (25 gf) and a holding time of 15 seconds, as outlined in prior studies.^23^^,^^24^

### Statistical analysis

The Kruskal–Wallis test was performed on the acoustic impedance and Vickers hardness values. Following the identification of significant differences between groups, the Steel–Dwass test was conducted to assess whether significant differences existed among the enamel and restorative materials. Measured Vickers hardness was compared with acoustic impedance values. After conducting a no-correlation test to ascertain the presence of correlation, simple correlation coefficients were utilised for evaluation. The significance level for all tests was set at *P* < .05.

Particularly concerning acoustic impedance values, emphasis was placed on the threshold for distinguishing between enamel and restorative materials. A statistical threshold based on the summation of standard deviations (σ₁ + σ₂) was employed to establish discriminability between materials. Furthermore, based on receiver operating characteristic (ROC) analysis, a discrimination threshold was determined to ensure practical clinical application. All statistical analyses were performed in Python (3.11, Anaconda distribution) and EXCEL Toukei Ver. 8.0.

## Results

### Ultrasound microscopic imaging

**Fig. 1:**
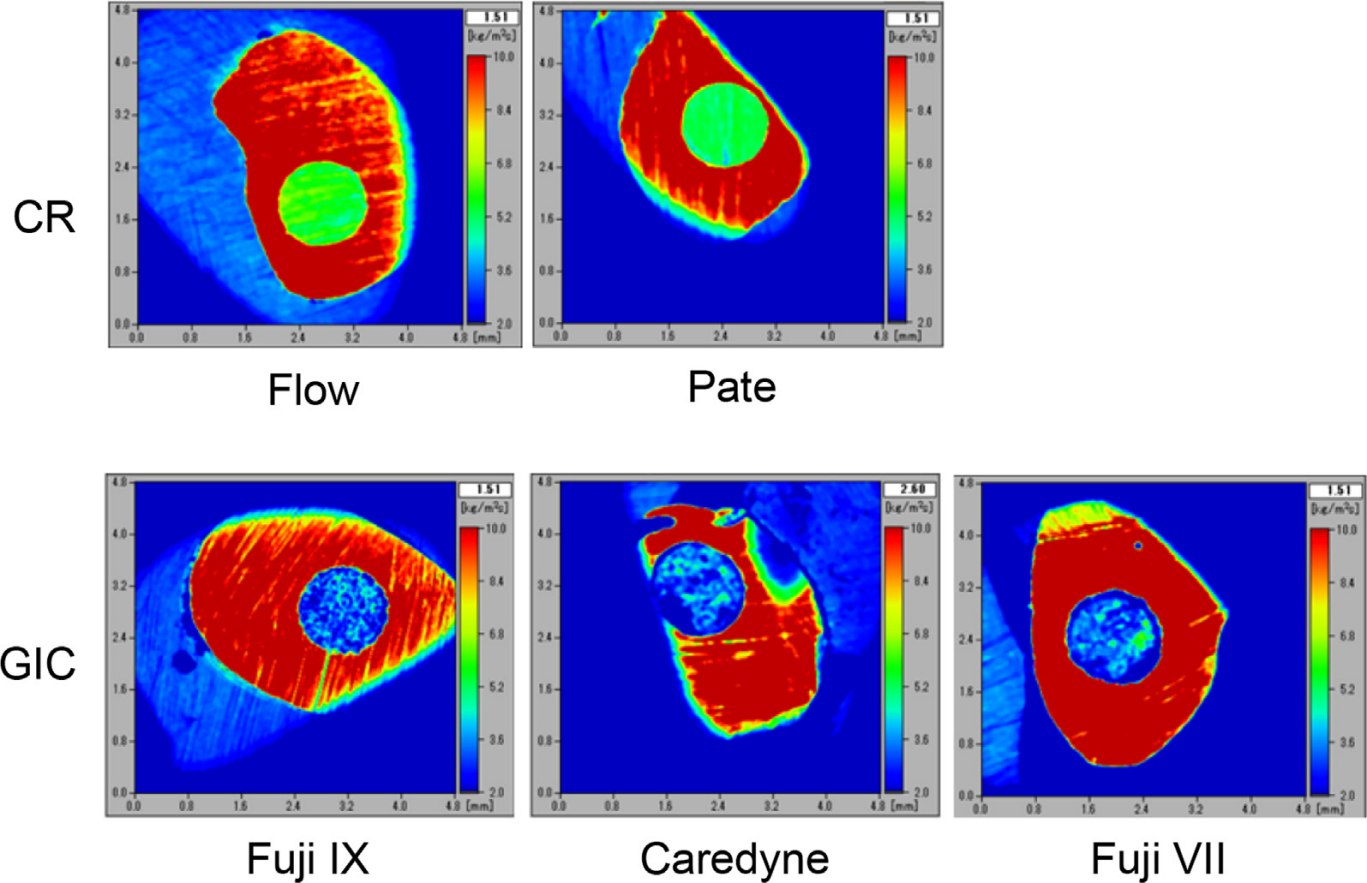
Ultrasound microscopic images of enamel and restorative materials. Images captured by ultrasonic microscopy are presented. The acquired acoustic impedance values are displayed as a colour image with 256 shades, ranging from 2.0 kg/m²s (blue) to 10.0 kg/m²s (red). Abbreviations: CR, composite resin; GIC, glass ionomer cement.

Figure 1 shows 2-dimensional colour mapping images obtained using an ultrasound microscope. Acoustic impedance was measured with an accuracy of 16 µm × 16 µm per pixel across an observation area of 4.8 mm × 4.8 mm, facilitating the generation of 2-dimensional colour images that distinctly distinguished between enamel and restorative materials. Enamel appeared prominently in red, attributable to its significantly elevated impedance compared to all restorative materials. The colour distribution for CR was uniform, while GIC displayed an uneven distribution across all samples.

### Comparison of acoustic impedance values among restorative materials

**Fig. 2:**
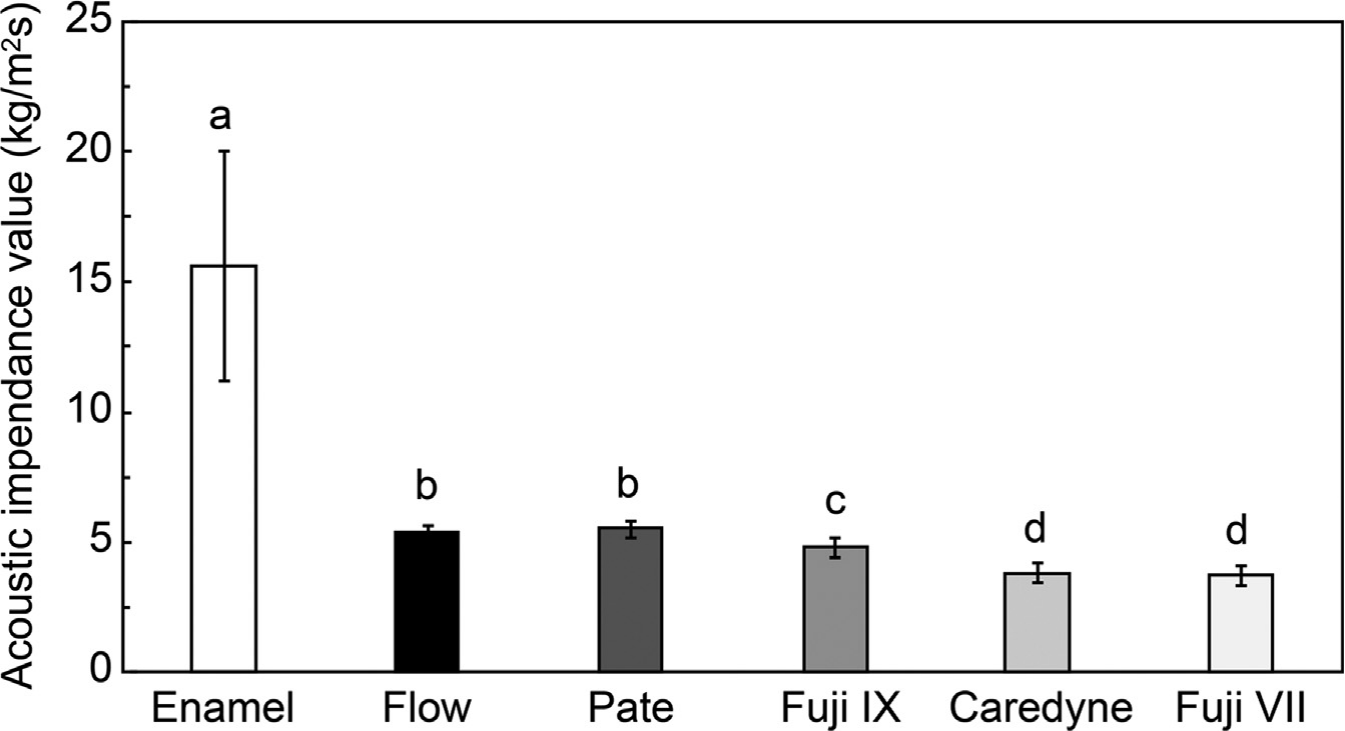
Comparison of the acoustic impedance values between enamel and restorative materials. The acoustic impedance values of enamel are significantly higher than those of all restorative materials. Among the restorative materials, CR exhibited significantly higher acoustic impedance values than GIC. Abbreviations: CR, composite resin; GIC, glass ionomer cement.

Figure 2 shows a comparison of the acoustic impedance values for enamel and restorative materials. The mean (± SD) acoustic-impedance value of enamel was significantly higher than that of all restorative materials. Table 1 summarizes the descriptive statistics. A Kruskal–Wallis test confirmed the overall differences among the 6 materials (*P* < .01), and Steel–Dwass post-hoc analysis identified 3 significant pairwise contrasts:

1. Enamel vs all restorative materials.
2. Flow- and Paste-type composite resins vs all glass-ionomer cements (GICs).
3. High-filler GIC vs multiple-ion and conventional GICs.

**Table 1:** Mean acoustic impedance values of enamel and restorative materials.

Material type	Mean acoustic impedance (kg/m²s) ± SD	Statistical significance
Enamel	15.6 ± 4.37	
Composite resin (Type flow)	5.36 ± 0.264	*P* < .01 vs enamel
Composite resin (Type paste)	5.49 ± 0.323	*P* < .01 vs enamel
Glass ionomer cement (High-filler)	4.80 ± 0.360	*P* < .01 vs enamel
Glass ionomer cement (Multiple ion)	3.80 ± 0.360	*P* < .01 vs enamel
Glass ionomer cement (Conventional)	3.74 ± 0.353	*P* < .01 vs enamel

Note: Pairwise comparisons confirm significant differences between the specified groups, as denoted in the table.

Consequently, the materials could be statistically clustered into 4 groups: (1) enamel, (2) Flow CR, (3) Paste CR, and (4) GICs.

For practical distinguishability, 2 complementary cut-offs were examined.

1. Statistical threshold–A pair was deemed distinguishable when the difference in their mean impedances exceeded the sum of their SDs (σ₁ + σ₂). Re-evaluating all pairs with this rule (Table 2) reproduced the 4 groups defined above and eliminated statistically significant yet clinically trivial overlaps (e.g., multiple-ion vs conventional GIC).

**Table 2:** Distinguishable pairs of materials are based on whether the difference in their mean acoustic impedance exceeds the summation of their respective standard deviations (σ₁ + σ₂).

Comparison	Mean hardness difference	Combined standard deviation	Distinguishability
Enamel vs type flow CR	10.24	4.634	Distinguishable
Type flow CR vs multiple ion type GIC	1.56	0.624	Distinguishable
Multiple ion type GIC vs Conventional type GIC	0.06	0.713	Not distinguishable

CR, composite resin; GIC, glass ionomer cement
2. Diagnostic threshold–ROC analysis identified an acoustic-impedance cut-off of 9.24 kg m⁻² s⁻¹, yielding 100% sensitivity and 100% specificity (area under the ROC curve [AUC] = 1.00). The corresponding positive likelihood ratio (LR⁺) was infinite, whereas the negative likelihood ratio (LR⁻) was zero. The positive predictive value (PPV) and negative predictive value (NPV) were both 1.00, confirming perfect diagnostic separation between enamel and restorative materials.

For comparison, applying the earlier statistical value of ≥ 15.6 kg m⁻² s⁻¹ to the same dataset preserved 100% specificity but reduced the sensitivity to 38% (LR⁻ = 0.62; NPV = 0.89), missing 16/26 enamel specimens.

Thus, the ROC-derived threshold is recommended for chair-side screening where false-negative results must be minimised, whereas the σ₁ + σ₂ rule remains useful for grouping materials in mechanistic or in-vitro analyses.

### Comparison of Vickers hardness between restorative materials

**Fig. 3:**
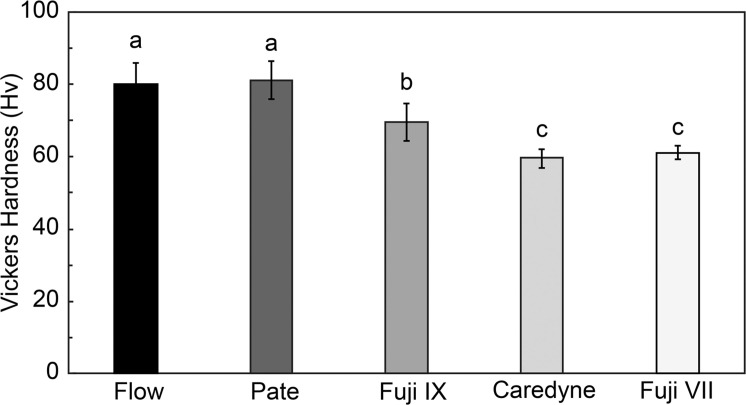
Comparison of the Vickers hardness values of the restorative materials. When comparing restorative materials, CR tends to have higher values than GIC. Among the restorative materials, no significant differences were observed among the types of CR. However, among the GICs, high-filler-type GICs exhibited significantly higher values than other GICs. Abbreviations: CR, composite resin; GIC, glass ionomer cement.

Figure 3 compares the Vickers hardness values of the restorative materials. When comparing the restorative materials, the Vickers hardness values tended to be higher in CR than in GIC, similar to the acoustic impedance values. No significant differences were observed between the CR types. Among the GIC types, the high-filler-type GIC (Fuji IX) exhibited a significantly higher Vickers hardness (*P* < .01).

### Relationship between acoustic impedance and Vickers hardness

**Fig. 4:**
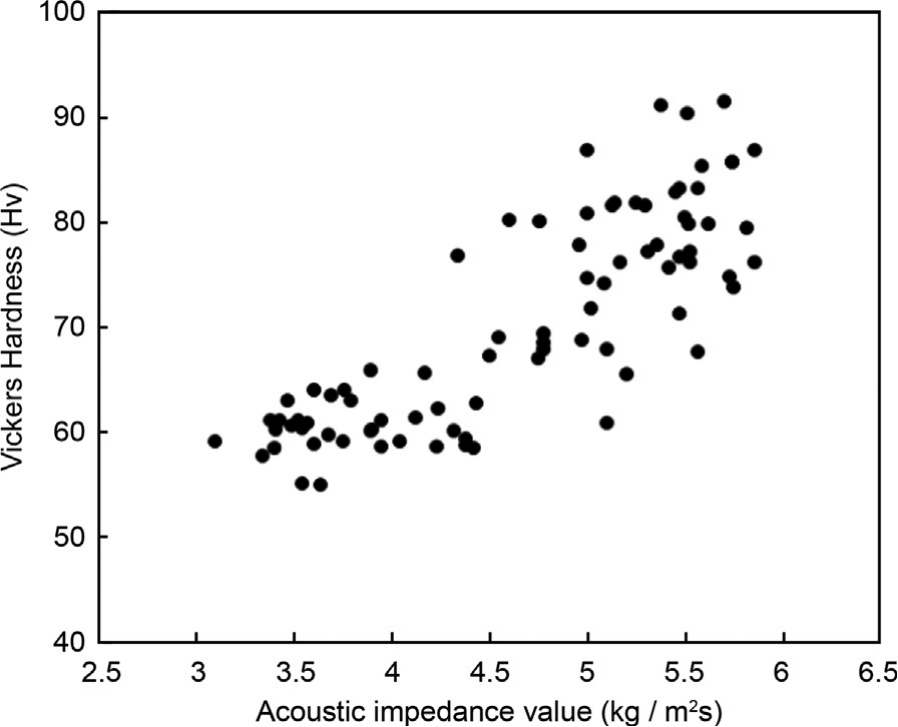
Relationship between the Vickers hardness and acoustic impedance values. A strong, positive, and significant correlation was observed between acoustic impedance and Vickers hardness.

Figure 4 shows the relationship between the acoustic impedance and Vickers hardness for all the restoration material samples. A strong and significant positive correlation was observed between acoustic impedance and Vickers hardness values (r = 0.831, *P* < .01).

**Fig. 5:**
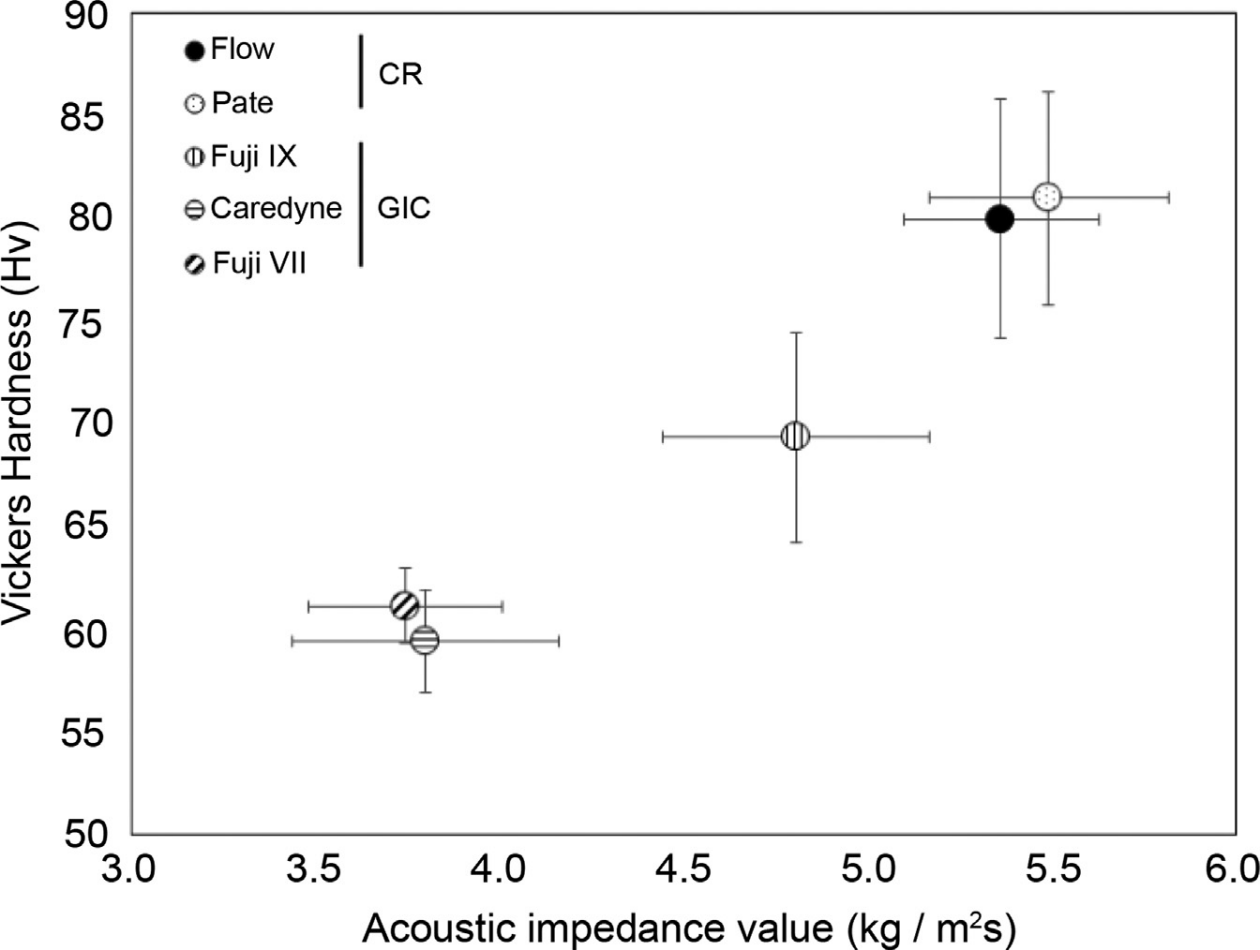
Relationship between acoustic impedance and Vickers hardness for each restorative material. The materials were broadly classified into 3 groups: conventional and multi-ion-releasing GICs, high-filler-type GICs, and CR. Abbreviations: CR, composite resin; GIC, glass ionomer cement.

Figure 5 shows the relationship between mean values of acoustic impedance and Vickers hardness for each restorative material. Samples were broadly divided into 3 groups: conventional GICs, multi-ion GIC, high-filler GIC, and CR.

## Discussion

The primary aim of this study was to investigate the potential of ultrasound microscopy as a chairside diagnostic tool during retreatment, by distinguishing enamel from existing CR and GIC based on their acoustic properties. Key findings demonstrated that the acoustic impedance of enamel was significantly higher than that of composite resins and glass ionomer cements, effectively allowing for the differentiation of these materials through 2-dimensional colour imaging techniques. Enamel exhibited a mean acoustic impedance of 15.6 ± 4.37 kg/m²s, compared to composite resins (Type flow: 5.36 ± 0.264 kg/m²s; Type paste: 5.49 ± 0.323 kg/m²s) and glass ionomer cements (Type high-filler: 4.80 ± 0.360 kg/m²s; Multiple ion: 3.80 ± 0.360 kg/m²s; Conventional: 3.74 ± 0.353 kg/m²s) (*P* < .01). These findings not only establish the feasibility of utilising ultrasound microscopy for material characterization in clinical settings but also address a critical gap in the literature regarding the acoustic evaluation of dental materials.

An ultrasound microscope serves as a tool for visualising and assessing the elastic properties of tissues based on measured acoustic parameters. The acoustic impedance was used as the acoustic parameter in this study. The relationship between acoustic impedance (z), sound velocity (c), density (ρ), and tissue elastic modulus (κ) can be expressed as κ *=* ρc^2^ = z^2^/ρ; thus, variations in acoustic parameters facilitate an objective measure of changes in the elastic modulus, that is, the hardness of substances.^25^^,^^26^ Ultrasound microscopy has been primarily employed for soft tissue diagnostics due to challenges related to the reflection of signals in hard tissues; it has also found applications in diagnosing diseases of the cardiovascular system and articular cartilage in clinical settings.^27^, ^28^, ^29^, ^30^ The clinical applications of ultrasound microscopy have evolved.^31^^,^^32^

Utilising ultrasound microscopy for tissue diagnostics offers the advantage of circumventing the need for sectioning and staining, whilst presenting acoustic parameters in a visually interpretable format.^14^^,^^33^, ^34^, ^35^, ^36^ Many studies have been performed on the application of ultrasound microscope in the field of dentistry.^37^, ^38^, ^39^, ^40^ The inside of hard tissues can now be measured noninvasively using ultrasound. However, as enamel constitutes a highly robust tissue with a high acoustic impedance value, 2-dimensional colour imaging has yet to be demonstrated, and no previous attempts have aimed to realize practical applications. Prior studies have indicated the potential of using ultrasound microscopy for evaluating the elastic properties of teeth through acoustic impedance assessment, showcasing considerable promise for diagnosing dental caries.^14^

This study revealed that the acoustic impedance of enamel was significantly higher than that of any of the restorative materials, thereby enabling a clear distinction between enamel and CR and GIC based on acoustic characteristics. Enamel, being the densest tissue in the human body predominantly composed of hydroxyapatite, exhibited a markedly higher acoustic impedance value relative to restorative materials.

Chemically, GICs are cured via an acid-base reaction. Hardened GICs have a core structure consisting of glass cores surrounded by a hydrogel (reaction product) embedded in a matrix.^4^^,^^41^^,^^42^ This structural characteristic may be reflected in the uneven mapping images of GIC obtained via ultrasound microscopy. When comparing GIC types, the high-filler GIC demonstrated higher acoustic impedance and hardness compared to the other types, as hardness generally increases in correlation with filler content.^22^

Contrarily, the imaging of CR revealed a uniform distribution in both flowable bulk-fill and paste types, which may be attributable to the following factors: both types are light-cured, there is no requirement for mixing, and the product is dispensed from a single paste, thus minimising manufacturing bias in component distribution. Furthermore, no significant differences in acoustic impedance or Vickers hardness were identified between the flowable and paste types. Based on manufacturer-provided data, no substantial discrepancies existed in mechanical properties among the cured restorative CR utilised in this investigation, thereby validating the findings obtained.

Upon comparing the restorative materials, the mapping images of GIC were found to be unevenly distributed for all materials, whereas the mapping images of CR were evenly distributed. This indicates that materials can indeed be distinguished based on their mapping image profiles. Both the Vickers hardness and acoustic impedance values were significantly higher in CR than in GIC, confirming that CR materials are generally more rigid than chemically cured GICs,^43^ consistent with our data. In this experiment, we observed a strong correlation between the Vickers hardness and the acoustic impedance values, suggesting that the acoustic impedance also reflected the hardness of the restorative materials.

In analysing the threshold for distinguishing between enamel and restorative materials, a statistical threshold based on the summation of standard deviations (σ₁ + σ₂) was utilised to establish discriminability between materials. However, this method does not account for diagnostic accuracy in clinical practice. To overcome this limitation, ROC analysis was conducted to ascertain a discriminability threshold optimised for 90% sensitivity and 95% specificity. Current chairside methods exhibit only moderate performance in distinguishing restoration margins. A recent *ex vivo* study reported sensitivities of 36% for conventional visual inspection and 97% when applying fluorescence-aided identification techniques (FIT).^13^ Using dentin shadow as the sole criterion, the same study demonstrated FOTI sensitivities of only 0% to 8%, compared with 56% to 69% for bite-wing radiography.^44^ In contrast, the present ultrasound approach achieved 90% sensitivity and 100% specificity for enamel–composite discrimination. Although direct head-to-head data are not yet available, these figures suggest a substantial diagnostic advantage. Future studies will therefore incorporate intraoral cameras, bite-wing radiographs, and FIT in a prospective, within-subject design to validate the clinical utility of ultrasound microscopy. Upon comparing the 2 methods, it was observed that while the statistical threshold proficiently categorised materials into distinct groups, the ROC-generated threshold offered a more clinically pertinent distinction, especially in differentiating between enamel and restorative materials.

### Limitations

This study had several limitations. First, it was conducted using *ex vivo* samples, which, despite careful preparation, may not fully recreate the intricate biological and physiological conditions present in vivo. Factors such as saliva, occlusal forces, and temperature fluctuations might influence material performance in clinical scenarios. Future investigations should encompass in vivo studies to enhance clinical relevance. Second, the exclusive use of human third molars as the sole sample limits the generalizability of our findings to other tooth types and broader populations. Although the *ex vivo* design eliminates clinical isolation challenges, the optical properties of anterior teeth–more relevant in esthetic zones–may vary from those of molars. To enhance clinical applicability, future studies should include a more diverse range of tooth specimens and patient demographics. Third, the lack of a validation cohort or independent dataset restricts the generalizability of the statistical findings. Although the Kruskal–Wallis and Steel–Dwass tests revealed significant differences among materials, external validation with an independent sample set or cross-validation methodologies remains essential to ensure robustness of the results. The study did not include a blinded visual-diagnostic arm. Consequently, direct head-to-head accuracy vs conventional inspection could not be assessed. Finally, while a statistical threshold for material distinction has been proposed, its clinical applicability necessitates further investigation. Receiver operating characteristics (ROC) analysis provides a data-driven cutoff value; however, its diagnostic accuracy in real-world scenarios requires validation. Future studies should explore testing the proposed threshold within clinical environments to evaluate its utility in guiding restorative material selection. To address these limitations, future studies should validate these findings using independent datasets, explore real-time assessment techniques, and conduct *in vivo* studies.

### Conclusions

Consequently, ultrasound microscopy serves a valuable role in distinguishing between enamel and restorative materials, as well as differentiating restorative materials (CR and GIC) through acoustic impedance measurement, thus underscoring the device's utility in dental practice. Compared to visual inspection, ultrasound microscopy demonstrates high inter-rater reliability due to its capacity to objectively and quantitatively measure hardness. Furthermore, it does not necessitate special protective measures owing to zero radiation exposure and offers the advantage of noninvasively measuring objects rapidly. Future endeavours will focus on developing a dental testing device utilising ultrasound to measure acoustic impedance intraorally, based on the data obtained in this study. The ultimate aim is to establish high-quality, standardised diagnostic methods that do not rely on subjective judgment or individual dentist skills.

## Declaration of competing interest

The authors declare that they have no known competing financial interests or personal relationships that could have appeared to influence the work reported in this paper.

## Appendix Supplementary materials

Image, application 1

## Footnotes

Supplementary material associated with this article can be found in the online version at doi:10.1016/j.identj.2025.100880.
