# Supplementary material for: Ultrasound Microscopy-Based Identification of Enamel and Restorative Materials: An Ex Vivo Acoustic Impedance Study
Source: Int Dent J. 2025 Jun 30;75(5):100880. doi: 10.1016/j.identj.2025.100880 (PMC12269967; doi:10.1016/j.identj.2025.100880)
Supplement: Supplementary file 1 [file mmc1.docx]

**SUPPLEMENTARY MATERIAL**


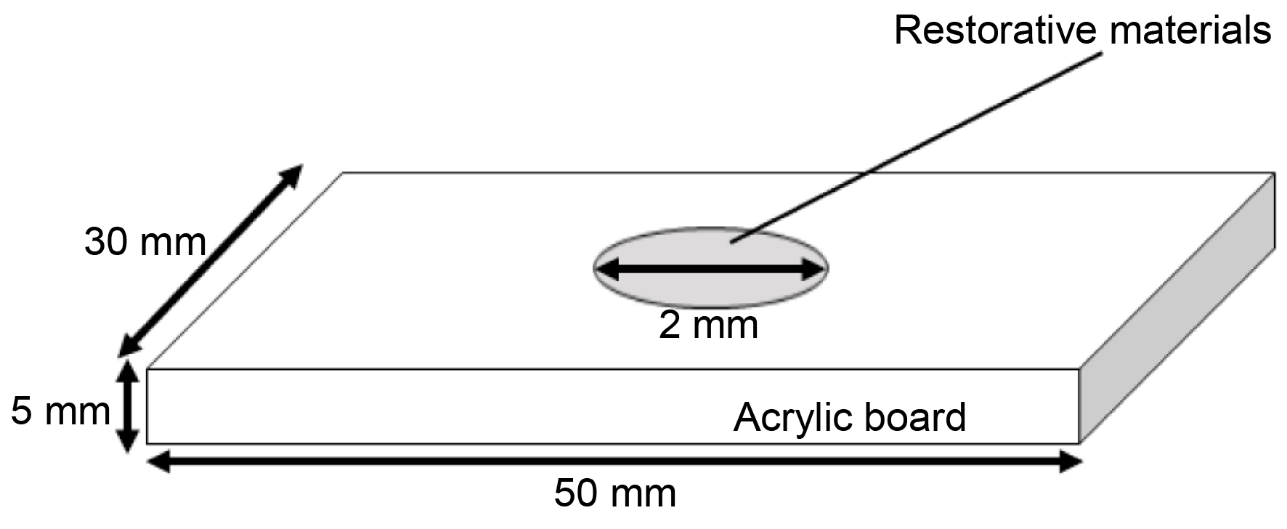


Figure S1：Schematic diagram demonstrating the acoustic impedance measurement of the restorative material samples

A cylindrical cavity with a depth of 3.0 mm was prepared in an acrylic plate measuring 30 mm × 50 mm × 5 mm, and a restorative material was filled into the cavity.


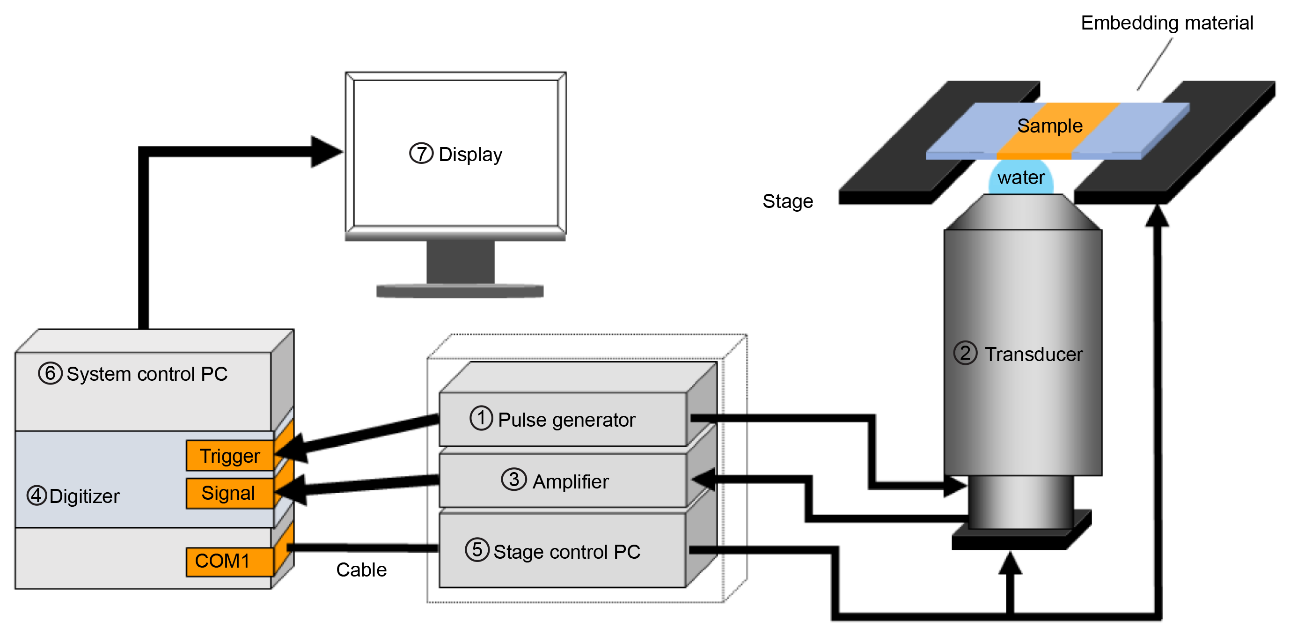


Figure S2: Block diagram of the ultrasound microscope system

This ultrasound microscope utilizes an ultrasonic transducer that performs two-dimensional scanning to non-invasively measure the acoustic parameters of tissue sections and cells at a microscopic level. The obtained information is then visualized as a high-resolution image on the display.


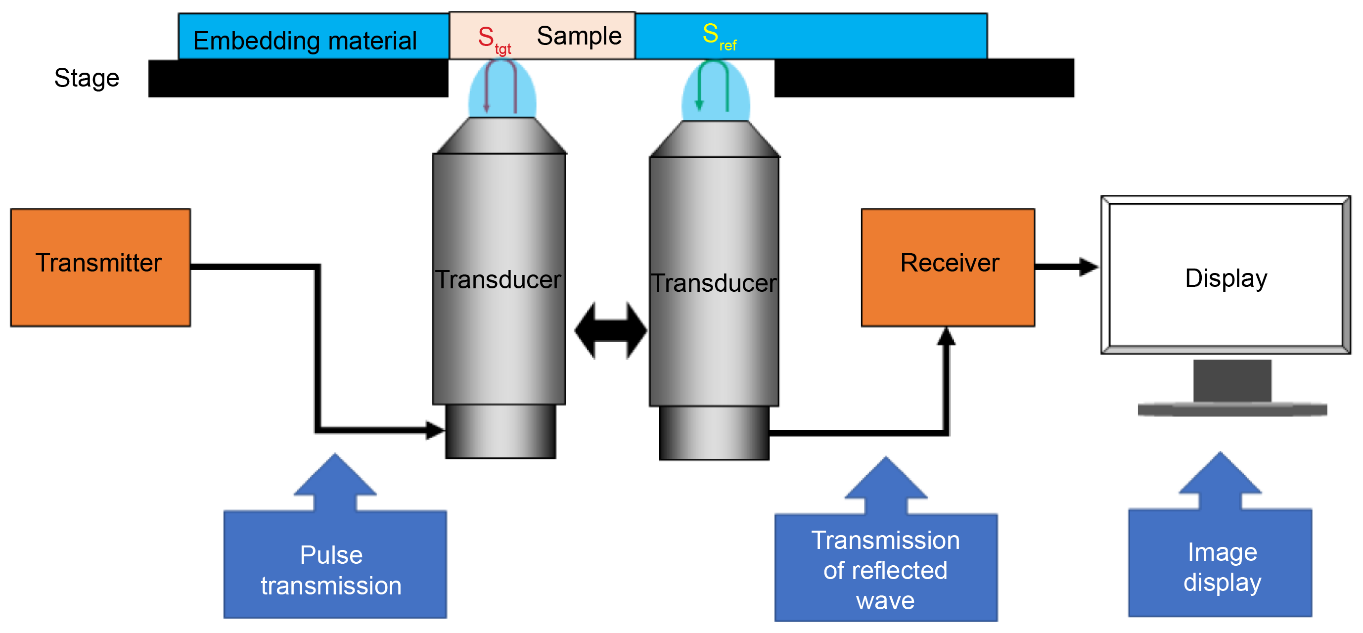


Figure S3: Enlarged view of the measurement unit

Stgt represents the reflected wave from the sample and Sref denotes the reflected wave component from the embedding medium.

Table S1: Restorative materials used in this study

| Category | Product name | Abbreviation | Type | Manufacturer |
| --- | --- | --- | --- | --- |
| CR | Gracefil Bulk Flow | Flow | Flow | GC |
|  | Gracefil Pate | Pate | Paste |  |
| GIC | Fuji IX GPEX | Fuji IX | High filler type |  |
|  | Caredyne | Caredyne | Multiple ion type |  |
|  | Fuji VII | Fuji VII | Conventional type |  |
